# EU postmarket surveillance plans for medical devices

**DOI:** 10.1002/pds.4859

**Published:** 2019-07-18

**Authors:** Josep Pane, Reynold D.C. Francisca, Katia M.C. Verhamme, Marcia Orozco, Hilde Viroux, Irene Rebollo, Miriam C.J.M. Sturkenboom

**Affiliations:** ^1^ Department of Medical Informatics Erasmus Medical Center – University Medical Center Rotterdam Rotterdam Netherlands; ^2^ Department of Patient Safety Alcon Fort Worth Texas; ^3^ Department of Global Health University Medical Center Utrecht Utrecht Netherlands; ^4^ Eu2P European Programme in Pharmacovigilance and Pharmacoepidemiology University of Bordeaux Segalen Bordeaux France; ^5^ Department of Regulatory Affairs HCL Technologies Frisco Texas; ^6^ Department of Patient Safety Novartis Barcelona Spain

**Keywords:** medical devices, pharmacoepidemiology, postmarket surveillance plan, risk management, safety evaluation

## Abstract

**Purpose:**

Recent public health safety issues involving medical devices have led to a growing demand to improve the current passive‐reactive postmarket surveillance (PMS) system. Various European Union (EU) national competent authorities have started to focus on strengthening the postmarket risk evaluation. As a consequence, the new EU medical device regulation was published; it includes the concept of a PMS Plan.

**Methods:**

This publication reviewed Annex III Technical Documentation on PMS and Annex XIV Part B: Postmarket clinical follow‐up from the new Regulation (EU) 2017/745 of the European Parliament and of the Council on medical devices.

**Results:**

The results of the PMS activities will be described in the PMS plan and will be used to update other related documents. A modular approach to structure the contents of the PMS plan will help to consistently update other PMS information. It is our suggestion that the PMS plan should consist of a PMS plan Core and a PMS plan Supplement. The PMS plan Core document will describe the PMS system, and the PMS plan Supplement will outline the specific activities performed by the manufacturer for a particular medical device.

**Conclusions:**

The PMS plan may serve as a thorough tool for the benefit‐risk evaluation of medical devices. If properly developed and implemented, it will function as a key player in the establishment of a new framework for proactive safety evaluation of medical devices.

**KEY POINTS**

- The new European Union (EU) postmarket surveillance (PMS) plan may serve as a thorough tool for the benefit-risk evaluation of medical devices.
- If properly developed and implemented, the EU PMS plan will function as a key player in the establishment of a new framework for proactive safety evaluation of medical devices.

## 1 INTRODUCTION

A medical device is defined as “any instrument, apparatus, appliance, material, or other article, whether used alone or in combination, including the software necessary for its proper application intended by the manufacturer to be used for human beings for the purpose of diagnosis, prevention, monitoring, treatment, or alleviation of disease, replacement, or modification of the anatomy or of a physiological process, and control of conception.”^1^ Medical devices are a great resource for enhanced diagnosis and disease management.

Recent public health safety issues involving medical devices have highlighted the need to update the European Union (EU) medical device regulation (MDR). The Poly Implant Prothèse (PIP) breast implant scandal in 2012 affected thousands of women and damaged the confidence of the different stakeholders involved in postmarket surveillance (PMS) of medical devices.^2^ More than 400 000 women around the world received PIP implants that were made of industrial‐grade silicone gel, prone to rupture, leading to inflammation and irritation. Another incident in 2012 involving hip implants raised a public health concern: metal‐on‐metal total hip replacements were successfully implanted, but metal abrading against metal caused erosion and leaching of metal particles into soft tissue.^3^ Such metal debris weakens tissue and bone around the implant, leading to implant failure, requiring additional surgery. The manufacturers did not provide an adequate response to the competent authorities with regard to these adverse events and there was always the belief that they could have been avoided.^4^

As a consequence, various national competent authorities (NCAs) and other health organizations started focusing on strengthening postmarket risk evaluation of medical devices. One of the important novelties in the new regulation on medical devices (EU) 2017/745, published May 5, 2017 is the concept of a PMS Plan for each medical device family.^5^ A regulation is a legal act of the EU that becomes immediately enforceable as law in all member states simultaneously. Regulations can be distinguished from directives which, at least in principle, need to be transposed into national law.^6^ The current Medical Device Directive (MDD) 93/42/EEC states that “The manufacturer shall institute and keep up to date a systematic procedure to review experience gained from devices in the post‐production phase, including the provisions referred in Annex X, and to implement appropriate means to apply any necessary corrective action.” Annex X says that “The clinical evaluation and its documentation must be actively updated with data obtained from the PMS. Where a postmarketing clinical follow‐up as part of the PMS plan for the device is not deemed necessary, this may be duly justified and documented.”^7^ Contrary to what happens with the new regulation, there are no instructions or guidance on the contents of the PMS plan and on how to implement this requirement in the current MDD 93/42/EEC although the concept of a PMS plan is mentioned.

According to the new regulation, the PMS Plan will have to define the process for collecting, recording, and investigating complaints and reports from healthcare professionals, patients, and users on events suspected to be related to a medical device. A PMS system that is correctly designed should allow for early detection of possible malfunctions and/or complications of medical devices that may occur only after years or even decades of usage and implement appropriate risk minimization measures.

Today, many medical device manufacturers have a “reactive” PMS system that is based on the collection of postmarket data received from spontaneous reporting of complaints and incidents. Unfortunately, there are few proactive PMS processes designed to actively gain knowledge on the safety and performance of the medical device through external sources like registries, electronic healthcare records, safety evaluation sites, claim databases, social networks, and literature.^8^

The new EU Regulation aims to reinforce key elements of the existing regulatory approach, including vigilance and market surveillance, at the same time ensuring transparency and traceability, to improve health and safety.^5^ The objective of this article is to describe the new EU Regulation on PMS of medical devices, to compare it with our experience in the drug area, and to provide recommendations for implementation.

## 2 PMS SYSTEM FOR MEDICINAL PRODUCTS AND MEDICAL DEVICES IN THE EU

### 2.1 Medicinal products

Manufacturers may submit a marketing authorization application to either European Medicines Agency (EMA) or to the NCAs of the member states. Authorization through the European Medicines Agency, also known as the centralized procedure, offers the benefit of a single assessment process and a marketing authorization valid throughout the European Economic Area. Authorization through the centralized procedure is mandatory for innovative medicines derived from biotechnology, orphan medicines, and new active substances for the treatment of acquired immunodeficiency syndrome, cancer, neurodegenerative diseases, diabetes mellitus, autoimmune diseases and other immune dysfunctions, and drugs targeting viral diseases.^9^

Similarly to medical devices, safety issues involving medicinal products showed a need for a more proactive risk management approach of medicinal products. This led to the development of the International Conference on Harmonisation of Technical Requirements for Registration of Pharmaceuticals for Human Use (ICH) E2E guidance on risk management planning. This guidance was implemented in EU regulation in 2005 in the form of the EU risk management plan (EU‐RMP), which is a mandatory template document for the authorization dossier of innovative drugs licensed in the EU.^10^, ^11^, ^12^ The EU‐RMP describes the important risks and areas of missing information, the activities intended to further characterize the safety profile, and the measures to minimize the risks.^13^, ^14^ The EU‐RMP is updated throughout the product life cycle as studies are completed or new information becomes available that may change the benefit‐risk balance.^15^ Significant variation exists in the requirements and execution of postauthorization safety studies (PASS) and additional risk minimization measures.^16^, ^17^, ^18^, ^19^ This is partly because the EU‐RMP is product‐specific and strategies are tailored to be risk‐proportionate (i.e. taking into account variables such as seriousness and severity of the risk, target population, and healthcare setting of use of the product).^20^ However, some variation is also due to marketing authorization holders: there is no gold standard for an optimal risk management organizational structure, and it depends on the magnitude and complexity of the company's pipeline, economic and staffing limitations, and organizational commitment to patient‐centeredness.^21^ Cross‐functional review of the risk minimization programs is recommendable and inclusion of senior management in final approval. The Pharmacovigilance Risk Assessment Committee (PRAC), an EMA scientific committee responsible for the review of all aspects of risk management planning, has been instrumental to overseeing postapproval commitments, and has played a key role in centralizing all the efforts to design and evaluate PASS .^22^ Table 1 describes some of the lessons learned from the pharmaceutical world and provides recommendations for implementation of the PMS plan for medical devices.

**Table 1:** Lessons learned from the pharmaceutical world and recommendations for implementation of the PMS plan for medical devices

Topic	Lessons Learned from the Pharmaceutical World	Recommendations for Implementation of the PMS Plan for Medical Devices
Enforcement of postapproval commitments	PRAC has played a key role to centralize all efforts to design and evaluate PASS; PRAC has been instrumental to enforce postapproval commitments related to PASS.	As part of the NB's oversight, there should be a centralized group responsible for monitoring and assessing the safety of medical devices. This group should include CA and notified bodies and should enforce the completion of CE mark commitments, such as postmarket studies or registries included in the postmarket clinical follow‐up plan.
Documentation, monitoring, and enforceability of postapproval commitments	Implementation of the EU‐RMP template triggered more proactive approaches and the documentation of many additional risk minimization activities. Enforceability of these postapproval commitments came from making these commitments conditions to the marketing authorization of the medicinal product.	Implementation of an actual PMS plan template is also important to document the postapproval commitments (e.g. postmarket studies and risk minimization activities). Enforceability of these postapproval commitments will come from making these commitments conditions to the marketing authorization of the medical device and verification during the annual PMS audits performed by the notified body.
Inclusion of risks in the PMS documents	Only important risks (risks that have an impact on the benefit‐risk balance) from the safety specification should be included into the PV plan.	Regulator‐led initiative to develop risk based approach guidances to recommend the inclusion of only important risks (risks that have an impact on the benefit‐risk balance) in the PMS documents (based on ISO 14971). Due to the wide range of medical devices and the different levels of complexity, these documents should be product‐specific.
Manufacturer's organizational adaptation	Cross‐functional review of the risk minimization programs and inclusion of senior management in final approval is recommended.	Cross‐functional review of the PMS plan is recommendable. The final approval of the PMS plan should be made by the PRRC within the company.

Abbreviations: EU, European Union; NB, notified body; PASS, postauthorization safety studies; PRAC, Pharmacovigilance Risk Assessment Committee; PMS, postmarket surveillance; PRRC, person responsible for regulatory compliance; RMP, risk management plan; PV, pharmacovigilance.

### 2.2 Medical devices

NCAs, notified bodies (NBs), and manufacturers are all involved in the European Conformity (CE) marking process that allows marketing of a medical device in the EU. The NB is an entity that has been accredited by an EU member state to assess whether a manufacturer's quality management system procedures and product technical documentation meets certain standards described in the EU MDD. With the NB's certificate, the manufacturer can then issue the declaration of conformity, and apply the CE Mark, which is required for sale in the EU. The conformity assessment can include inspection and examination of a product, its design, and the manufacturing environment and processes associated with it, including the safety evaluation of the medical device.

NCA's exist in each European member state and are nominated by each government to monitor and ensure compliance with its provisions of the MDD 93/42/EEC. The NCA designates a NB to ensure that conformity assessment procedures are completed according to the relevant criteria. The authorized representative, designated by the manufacturers (there is only an authorized representative when the manufacturer is not based in the EU; when the manufacturer is based in the EU, the manufacturer is the direct point of contact), is legally responsible for compliance with the regulations and acts as the first point of contact for the EU authorities. It is the manufacturer's responsibility to ensure that their product complies with the essential requirements of the relevant EU legislation. Medical devices are classified based on the risk associated with them, using the classification rules listed in Directive 93/42/EEC Annex IX. The categories are Class I, Class IIa and IIb, and Class III, with Class III ranked as the highest. The higher the classification, the greater the level of assessment required by NBs. The classification is based on the intended purpose of the device and not the particular technical characteristics. There are different aspects that are being taken into consideration for classification: grade of invasiveness, duration of contact with the body, and local versus systemic effect.^7^, ^23^

In order to obtain the CE mark that allows marketing of a medical device in the EU,^24^ the manufacturer is obliged to identify and describe the risks detected during the pre‐market phase.^1^, ^5^ The risk management file (RMF) of the medical device or its family should contain clear definitions of the hazardous situations associated with use of the medical device. In addition, it should also describe the potential harms associated with these situations as well as the applicable risk minimization measures to avoid or mitigate these harms in both patients and healthcare users.

According to the new EU MDR for medical devices, a comprehensive RMF demonstrating a positive benefit/risk profile is conditional to marketing and required to be monitored postmarketing in a timely manner. The new EU MDR has additional requirements in PMS and Vigilance compared with the current MDD (Tables 2 and 3). The new EU MDR states that the PMS plan “shall be suited to the actively and systematically gathering, recording and analysing relevant data on the quality, performance and safety of a device throughout its entire lifetime, and to drawing the necessary conclusions and to determining, implementing and monitoring any preventive and corrective actions”.^5^ Table 4 specifies the main technical requirements of the PMS plan. The final approval of the PMS plan should be made by the person responsible for regulatory compliance (PRRC) within the company.

**Table 2:** PMS System: comparison between the current MDD^7^ vs the new MDR^5^

MDD PMS Key Principles	MDR Additional PMS Requirements Compared with MDD
Systematic procedure to review experience gained from the market.	PMS oversight: notified bodies and competent authorities have increased postmarket surveillance authority for unannounced audits, samples checks, and annual safety reports.
Obligation to report incidents and increase in trends.	Clinical Evidence: Manufacturers need to conduct clinical investigations and collect postmarket clinical data as part of ongoing safety assessment.
	PMCF plan to be part of the PMS plan. One PMS plan and one PSUR per device/device group/family.

Abbreviations: MDD, Medical Device Directive; MDR, medical device regulation; PMCF, the postmarket clinical follow‐up; PMS, postmarket surveillance; PSUR, periodic safety update report.

**Table 3:** Medical device vigilance system: comparison between Meddev 2.12‐1^25^ vs the new MDR^5^

Topic	Meddev 2.12‐1	MDR
What to report?	• Near incident (serious)	• Serious incidents
	• Serious incident	
Reporting timelines	• Serious public health threat: 2 days	• Serious public health threat: 2 days
	• Death or unanticipated serious deterioration in state of health: 10 days	
		• Death or unanticipated serious deterioration in state of health: 10 days
	• Other reportable incidents: 30 days	
		• Other serious incidents: 15 days
Periodic summary reports	When agreed with the coordinating CA:	When agreed with the coordinating CA:
	• For similar incidents with known root cause or FSCA implemented	• For similar incidents with known root cause or FSCA implemented
	• For common, well documented incidents	
		• For common, well‐documented incidents
Report to	• NCA	• Centralized electronic reporting in EUDAMED
Trend reporting	Trend reporting is used by the manufacturer when a significant increase in events not normally considered to be incidents and for which predefined trigger levels are used to determine the threshold for reporting.	Mandatory reporting of:
		• Statistically significant increase in frequency or severity of non‐serious incidents or expected side‐effect that could impact risk/benefit ratio
		• ‘statistically significant increase’ needs to be defined upfront in the Tech File as part of the PMS plan for the device
		The EU Commission will perform trending and signal detection based on the data in Eudamed.
FSCA	• The details of FSCAs are communicated by manufacturers to the NCAs via FSCA form and to the users in FSNs.	• The details of FSCAs are communicated by manufacturers to the NCAs via FSCA form and to the users in FSNs.
		• The NCA may perform their own risk assessment, manufacturer has to provide the supporting documentation.
		• The NCA may intervene in the manufacturer's investigation.
		• The FSN needs to contain the UDI and the manufacturer's SRN and needs to be uploaded in Eudamed.
		NCAs may ask manufacturers for corrective actions and will inform the NB, other manufacturers and the EU Commission.
PSUR	Not included in the current guideline.	• Class I devices: PMS report updated when necessary, but at least every 5 years.
		• Class IIa: PSUR to be updated when necessary, but at least every 2 years.
		• Class IIb (non‐implantables): PSUR to be updated annually.
		• Class IIb (implantables), III: PSUR to be updated annually and sent to the NB for evaluation.
		• Analysis of PMS data.
		• Description of preventive and corrective actions.
		• Conclusion of the benefit/risk evaluation.
		• Main findings of the PMCF report.
		• Sales volumes, estimate of the population using the device, usage frequency of the device.

Abbreviations: CA, competent authority; EU, European Union; EUDAMED, European Database on Medical Devices; FSCA, field safety corrective action; FSN, field safety notice; MDR, medical device regulation; NB, notified body; NCA, national competent authority; PMCF, postmarket clinical follow‐up; PSUR, periodic safety update report; UDI, unique device identifier.

**Table 4:** Essential requirements from the EU regulation for medical devices that are relevant to the technical documentation on postmarket surveillance – Extract of the EU regulation.^5^

EU MDR (Annex III Technical Documentation on Postmarket Surveillance):
The manufacturer shall prove in a postmarket surveillance plan that it complies with the obligation referred to in Article 83
(a) The postmarket surveillance plan shall address the collection and utilization of available information, in particular:
‐ Information concerning serious incidents, including information from PSURs, and FSCAs;
‐ Records referring to non‐serious incidents and data on any undesirable side‐effects;
‐ Information from trend reporting;
‐ Relevant specialist or technical literature, database and/or registers;
‐ Information, including feedbacks and complaints, provided by users, distributors, and importers; and
‐ Publicly available information about similar medical devices;
(b) The postmarket surveillance plan shall include at least:
‐ A proactive and systematic process to collect any information referred to in point (a). The process shall allow a correct characterization of the performance of the devices and shall also allow a comparison to be made between the device and similar products available on the market;
‐ Effective and appropriate methods and processes to assess the collected data;
‐ Suitable indicators and threshold values that shall be used in the continuous reassessment of the risk benefit analysis and of the risk management as referred to in Section 3 of Annex I;
‐ Effective and appropriate methods and tools to investigate complaints or market experiences collected in the field;
‐ Methods and protocols to manage the events subject to trend report as provided for in Article 88, including the methods and protocols to be used to establish any statistically significant increase in the frequency or severity of incidents as well as the observation period;
‐ Methods and protocols to communicate effectively with competent authorities, notified bodies, economic operators, and users;
‐ Reference to procedures to fulfil the manufacturers obligations laid down in Articles 83, 84, and 86;
‐ Systematic procedures to identify and initiate appropriate measures including corrective actions;
‐ Effective tools to trace and identify devices for which corrective actions might be necessary; and
‐ A PMCF plan according to in Part B of Annex XIV, or a justification why a PMCF is not applicable.

Abbreviations: FSCA, field safety corrective action; PMCF, postmarket clinical follow‐up; PSUR, periodic safety update report.

**Figure 1:**
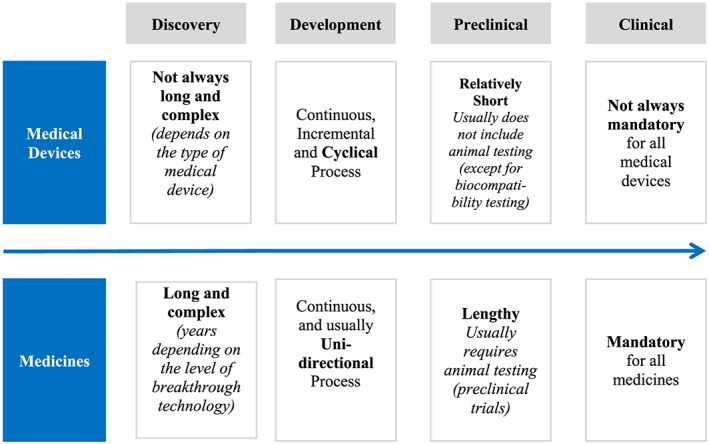
Overview of the main differences during new product development between medical devices and medicines [Colour figure can be viewed at wileyonlinelibrary.com]

**Figure 2:**
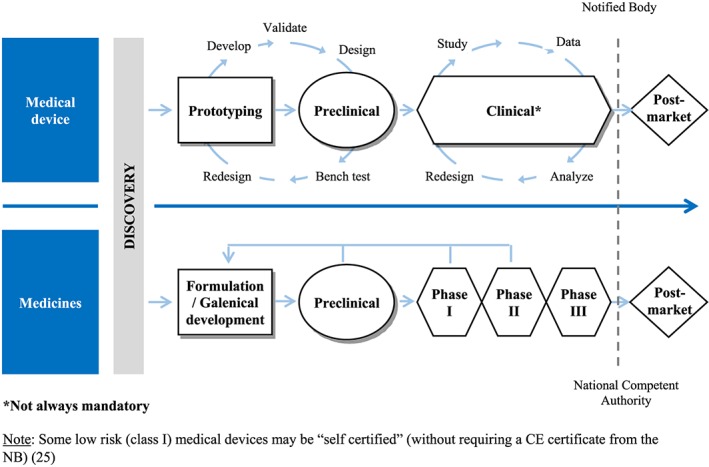
The medicinal product and the medical device development pathway in the EU [Colour figure can be viewed at wileyonlinelibrary.com]

**Figure 3:**
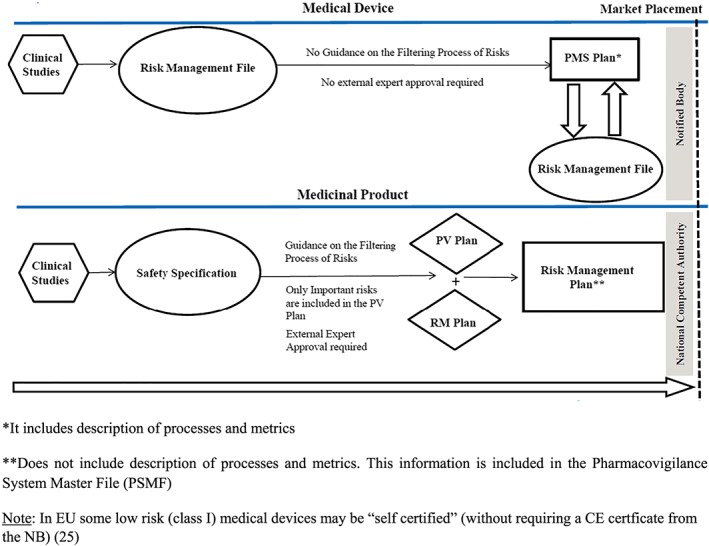
Risk managements documents required for the market placement of a medical device compared with a medicinal product [Colour figure can be viewed at wileyonlinelibrary.com]

To understand the key differences between the flow of risk management documents for a medical device and a medicinal product, it is important to understand the main differences between medical devices and medicines during new product development (Figure 1) and the main differences during the development pathway (Figure 2).^8^ Figure 3 describes the flow of risk management documents that are required for a medical device and a medicinal product. One of the key differences between the two products is the filtering performed for medicinal products: only important risks (risks that have an impact on the benefit‐risk balance) from the safety specification should be included into the pharmacovigilance (PV) plan. For medical devices, there are no regulatory documents that provide guidance on filtering the risks from the RMF into the PMS plan. The RMF of a medical device includes the risk analysis, the risk evaluation, the implementation and verification of the risk control measures, and the assessment of the acceptability of any residual risk. Another difference with regard to medical devices is that the RMP of a medicinal product needs to be reviewed and approved by regulatory authorities, whereas the RMF or the PMS plan of a medical device are reviewed by the NB and do not require approval from the NCA. Contrary to what happens with medicinal products where the process goes through the EMA, or the designated NCA, in EU, the medical devices do not need to be approved by the NCA. In EU, the new medical device application (if required) is performed by the NB—an entity that examines the medical device application to assure compliance with the EU regulation. If the device meets regulatory requirements, a CE is applied, and the medical device can be marketed throughout Europe.^26^

## 3 RECOMMENDATIONS FOR IMPLEMENTATION OF THE PMS PLAN FOR MEDICAL DEVICES

Most of the current PMS requirements are included in the medical device guidelines, and not in the current MDD; this has led to enforcement challenges for the manufacturer's requirements. With the new regulation, the EU wanted to eliminate those challenges and, at the same time, provide instructions on how to build a more proactive PMS system (Tables 2 and 3).

Based on the requirements described in the new regulation and the lessons learned from medicinal products, we would like to propose the following recommendations for implementation of the new legislation. We have designed a template for the PMS plan content (see Tables 5 and 6). The PMS plan becomes a master file and consists of a PMS plan Core (Table 5) and a PMS plan Supplement (Table 6) containing different modules of PMS data. The Core document should describe the PMS system (routine PMS procedures, methodologies, and activities that are being performed for all medical devices or group/family of medical devices) as well as the key performance indicators (KPIs) used to evaluate the effectiveness of the plan. The Supplement should describe the specific PMS activities, methodologies, and procedures performed by the manufacturer for a particular medical device or family/group of medical devices. The PMS Plan shall also define the frequency of the PMS data review. The manufacturer should institute a system to assess all the PMS information with a specific frequency and implement the necessary actions to improve safety and performance of the product. The Core and the Supplement should have different review timelines: the PMS plan Core only describes the processes and does not require a continuous update of the content. The periodicity of renewal of the PMS plan Supplement should be consistent with the risk associated to the product, the innovative character of the device, and the level of clinical experience with the device. For example, as a general rule, classes IIb and III medical devices should be reviewed on a yearly basis and class IIa on a biannual basis (*Note*. Class I devices still need a review, but it is a simplified PMS supplement that should be updated at least every 5 years).

**Table 5:** Suggested template: PMS plan Core

1. PMS	
Data source	All data source for that medical device
Complaint management (this would be part of the processes subsection)	Intake of an adverse event/technical complaint
	Medical review
	QA product investigations
	Follow‐up
	Submission/reporting process
Customer feedback (subsection of source data)	Postmarket clinical follow‐up plan (subsection of processes)
Monitoring of product benefit‐risk profile (subsection of processes)	Adverse event trending
	Technical complaint trending
	Postproduction information
Risk management (subsection of processes)	Field action assessment committee
	Device medical safety review board
	Safety governance review board
2. Risk minimization measures (part of the risk management)	
Communication of safety concerns	Safety communication process
Effectiveness of risk minimization measures	Risk reduction process
Labeling committee	Labeling risk minimization measures
3. Other PMS‐related processes and key SOPs	

Abbreviation: PMS, postmarket surveillance; QA, quality assurance; SOP, standard operating procedure.

**Table 6:** Suggested template: PMS plan Supplement

1. Product Overview		
Product name(s)/family		
Approved indication(s)		
Population being treated		
Medical device risk classification		
License partners (if applicable)		
2. Summary of Safety Concerns		
Safety Concern	Hazard	Harm
Important identified risks		
Important potential risks		
Missing information		
3. Risk Minimization Measures		
Inherent safety by design and construction		
Protective measures in the medical device itself or in the manufacturing process		
Training to users and/or information for safe and proper use		
Conduct of a study		
Communication of a FSCA		
4. Additional PMS Activities		
Activity	Rationale	
	
	
	
	
5. Plans for PMCF and Clinical Evaluation		
Summary of PMCF report (including registry review) and CER		
6. Safety Communications		
External and internal communication of safety concerns		
7. Annexes		
Training of Personnel		
Documents and Records		
8. References		

Abbreviations: CER, clinical evaluation report; FSCA, field safety corrective action; PMCF, postmarket clinical follow‐up; PMS, postmarket surveillance.

The final approval of the PMS plan should be made by the PRRC. However, the PMS plan should also define who will review the PMS plan. We have learned in the drug era that the manufacturers should create an organizational model that ensures an efficient cross‐functional review and senior management communication and the systematic incorporation of patient and healthcare professionals input into the PMS workflow. Key individuals from the different departments such as Medical Safety, Clinical, Research and Development, Regulatory Affairs, Compliance and Quality Assurance should participate in the production of the Core and Supplemental PMS plan. The final review of the documents should be performed by a cross‐functional senior management team.

Prior to launch, the manufacturer shall incorporate the risk minimization measures. The actual PMS plan and the activities involved with it may also lead to risk minimization measures such as a change in the labeling, a design change, or a material change. The new risk minimization measure will need to be documented in a consistent and timely manner across the other PMS documents (such as Risk Management and Periodic Safety Update Reports). This will be ensured by the use of the suggested modular approach (see Table 6) for the PMS plan structure.

A program of appropriate PMS including postmarket studies and registries is very important to detect and investigate risks associated with the use of marketed medical devices and should be included in the postmarket clinical follow‐up (PMCF) plan. The plan describes methods for clinical data collection to confirm the safety and performance of a device throughout its lifetime; these methods may include postmarket studies or registries as appropriate.

Postmarket studies and registries provide information on “real world” use and are a component of PMS. The postmarket studies can be sponsor‐led (sponsored by the manufacturer) or investigator‐initiated trials (IITs) which are any scientific study, other than a manufacturer‐sponsored study, originated and proposed by a third party investigator. Medical device registries can be sponsor‐led or health authority‐mandated and are designed for different purposes. They can offer valuable data on long‐term effectiveness and safety of devices or on the impact of factors such as surgical method, physician, hospital, and patient conditions.^27^

It is important to take into consideration that data from these studies and registries need to be used for continuous evaluation of the benefit‐risk profile as well as for discovery of new indications of use. When the PMCF study is completed, there should be a final report with clear conclusions that will be included in the periodic safety update report (PSUR).

**Figure 4:**
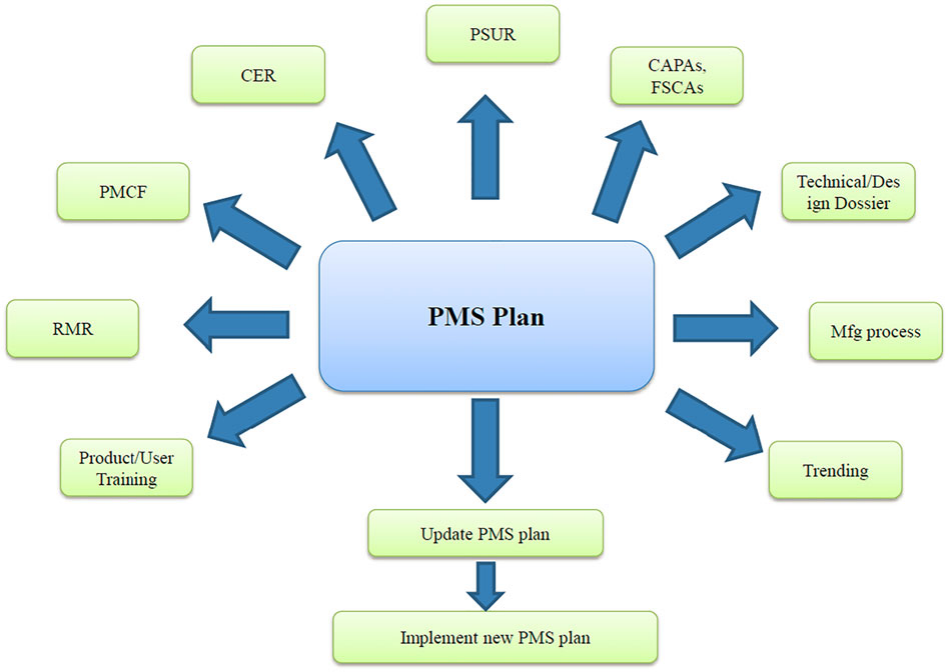
Output of the postmarket surveillance (PMS) plan [Colour figure can be viewed at wileyonlinelibrary.com]

The results of PMS activities will have an impact on the PMS process during the device life cycle management. Some of the information from the PMS plan will be used to update other related PMS documents. A modular approach to structure the contents of the PMS plan may help to consistently update other PMS information. The output of the PMS plan could lead/affect different postmarket documents (Figure 4). For example, after the review of national registries (part of the PMCF up plan), the manufacturer may identify a new safety issue with the product that will affect different postmarket documents: update of RMR, update of clinical evaluation report (CER), new PSUR, development of corrective and preventive actions (CAPAs), new training to the user, or submit a field safety corrective action (FSCA) to the NCA.

To measure the effectiveness of the PMS plan, it is important to have adequate tools in place for each of the processes. KPIs must be identified a priori when building the processes. Moreover, together with the KPIs, it is essential to identify a threshold for each of the indicators to take action if this threshold is reached. Therefore, the key processes that need to be measured should be identified, and the significant points of measurement that define the performance of the systems should be described in the PMS plan. These measures will help to identify areas of improvement. In Table 7, we propose different KPIs to monitor the performance of the PMS system, there should be KPIs for case processing, safety communications, PSURs, risk management, early detection of signals, and implementation of corrective actions.

**Table 7:** Proposed KPIs to measure effectiveness of PMS plan

Process	KPI	Type	
		Quality	Timeliness
1. Case processing	Expedited reporting on time	‐	✓
	Periodic Reporting on time	‐	✓
2. Case quality review	Case quality review	✓	‐
	Quality review of regulatory reports	✓	‐
	Comments and Inquiries received from CA after the submission of a regulatory report	✓	‐
3. Periodic search of scientific literature	Literature search review timeliness	‐	✓
	Peer review of selected abstracts	✓	‐
	Peer review of rejected abstracts	✓	‐
4. Aggregate reports	PSUR submission timeliness to CAs	‐	✓
	Comments and Inquiries received from CA after the submission of PSUR	✓	‐
5. Safety communications	Safety communications submitted on time	‐	✓
	Comments and inquiries from CAs, healthcare professionals, or consumers received after the submission of the safety communications	✓	‐
6. Signal detection	Signals detected on time; timely identification of safety issues	‐	✓
	Signal evaluation and validation performed effectively; real signal?	✓	‐
7. Corrective action	Corrective actions implemented on time	‐	✓
	Corrective actions effectiveness	✓	‐
8. Risk management	Risk management file timely review; timely update of the risk management file	‐	✓
	Rates of comments and inquiries from CAs by impact	✓	‐

Abbreviations: CA, competent authority; KPI, key performance indicator; PMS, postmarket surveillance; PSUR; periodic safety update report.

## 4 DISCUSSION

This paper tries to provide implementation guidance to the medical device EU‐regulation based on lessons learned from the medical product area. We have seen how vital it is to identify the risks in a timely manner for all stakeholders to be aware of the risks associated with medical devices. Stakeholders need to take appropriate corrective and preventive measures to improve patient outcome^3^ resulting in a device that is safe and performs well.

We conclude that the PMS plan needs to include the identified risks, potential risks, and missing information from the RMF. Next, safety evaluation tools (CER, PSUR, RMF) to find responses to unanswered questions and find more information regarding missing information should be implemented. The PMS plan should have clear objectives, a robust structure with specifications on data integrity, periodicity, and defined responsibilities. We recommend a modular approach to structure the contents of the PMS plan that will facilitate consistent updating of other PMS information. The PMS plan should consist of a PMS plan Core and a PMS plan Supplement. The PMS plan Core document will describe the manufacturer's general PMS system, and the PMS plan Supplement will describe the specific PMS activities performed by the manufacturer for a particular medical device or family/group of medical devices. Since we learned from the medicinal products area that a template is important, we proposed one. In addition to the template, another important aspect learned from the experience with medicinal products is the methodology used to include customer feedback and the organizational structure within the company. To deliver high‐quality PMS plans, companies need to implement a system that includes cross‐functional review and takes into account the patient feedback received during the postmarket phase. A difference with medicinal products is the fact that no filtering is implemented: we would recommend that the regulatory bodies develop product‐specific guiding documents outlining how to perform the filtering of risks from the RMF to the PMS plan and also provide guidance on the stakeholder responsibility in reviewing and approving the PMS plan.

Moreover, to ensure the success of the PMS plans, the manufacturers should first identify the key processes of the plan and define KPIs as well as the associated thresholds to take action. These indicators will help to measure the effectiveness of the plan.

In conclusion, the new EU MDR may positively impact medical device safety evaluations and calls for a more hands‐on approach, which does not only consist of spontaneous reporting, but also includes proactive methods to manage product‐related risks with new safety evaluation tools such as the PMS plan. There are several questions regarding the implementation of the new EU medical device guideline and differences with medicinal products. This paper tries to review them and provide some guidance.

## ETHICS STATEMENT

The authors state that no ethical approval was needed.

## CONFLICT OF INTEREST

Josep Pane and Marcia Orozco are employees of Alcon, which manufactures medical devices. Irene Rebollo is an employee of Novartis, which manufactures medical devices. Hilde Viroux is an employee of HCL Technologies, an engineering services company, active in many areas including medical devices. Reynold Francisca, Katia Verhamme, and Miriam Sturkenboom have no conflicts of interest that are directly relevant to the content of this manuscript.

## DISCLAIMER

The views expressed in this article are the personal views of the author(s) and may not be understood or quoted as being made on behalf of or reflecting the position of Alcon, Novartis, Erasmus Medical Center or University Medical Center Utrecht, HCL Technologies, or one of its committees or working parties.

## FUNDING INFORMATION

No sources of funding were used to assist in the preparation of this study.
